# Elimination disorders and associated factors among children and adolescents age 5–14 year-old attending paediatric outpatient clinic at Wolaita Sodo University comprehensive specialized hospital, South Ethiopia

**DOI:** 10.1186/s13034-024-00739-7

**Published:** 2024-05-03

**Authors:** Tamene Berhanu, Mubarek Abera, Shimelis Girma, Yonas Tesfaye

**Affiliations:** 1 Wolaita Sodo University College of Health Science and Medicine, Wolaita Sodo, Ethiopia; 2 Department of Psychiatry, Faculty of Medical Sciences, Jimma University, Jimma, Ethiopia

**Keywords:** Elimination disorder, Enuresis, Encopresis, Combined elimination disorder, Children, Adolescents, Wolaita Sodo, South Ethiopia

## Abstract

**Background:**

Elimination disorder occurs in children over the age of normal toileting who continue to have an inability to control urination or feces, either during the day, at night, or both. Paediatric elimination disorders are not well understood by parents, teachers, medical professionals, mental health practitioners, and researchers. Hence, this study aimed to assess the magnitude of elimination disorder and associated factors among children and Adolescents aged 5–14 years old at Wolaita Sodo University Comprehensive Specialized Hospital, South Ethiopia, in 2022.

**Method:**

A hospital-based cross-sectional study was conducted from September 22 to November 22, 2022, at Wolaita Sodo University Comprehensive Specialized Hospital. A systematic random sampling technique was employed to select 423 study subjects. The data were gathered using a structured, face-to-face interviewer-administered questionnaire. The development of the symptom score for dysfunctional elimination syndrome of Vancouver questionnaires was used to screen for elimination disorders. Logistic regression model was used to determine the association between the outcome and independent variables. A 95% CI and Odds ratio with corresponding p-value < 0.05 were used to determine the predictors of the outcome variable.

**Result:**

The overall magnitude of elimination disorder among children and Adolescents age 5–14 in this study was (n 70, 16.8%); in boys (n 47, 17.3%) and girls (n 23, 15.75%). The prevalence of enuresis was (n 64, 15.3%), encopresis (n 15, 3.6%), both enuresis and encopresis, or combined elimination disorder (n 9, 2.2%). Age 9–11 years (AOR = 3.2, 95%CI:1.09, 9.43), family size four and above (AOR = 3.4, 95%CI:1.78, 6.56), family history of elimination disorder (AOR = 3.9, 95%CI:2.12, 7.45), emotional problem (AOR = 2.2, 95%CI:1.18, 4.05), hyperactive problem (AOR = 3.8, 95%CI:1.83, 7.83), low toilet training skills (AOR = 5.9, 95%CI:2.61, 13.33), bad parenting practices, were poor supervision (AOR = 4.4, 95%CI 1.29, 14.69) were significantly associated with elimination disorder.

**Conclusion and recommendation:**

In this study, approximately one in five children and adolescents had an elimination disorder. Younger age, family size four and above, positive family history of elimination disorder, presence of emotional and hyperactive problems, bad parenting practices, and low toilet training skills were factors associated with elimination disorders. Therefore, preventative, etiological, and therapeutic measure, early toilet training, supportive parenting practices, screening for children’s and adolescents’ behavioral problems, and elimination disorders need attention to reduce the effect of the problem.

## Introduction

Elimination disorders (ED) include enuresis, the repeated passing of urine during the day or night into inappropriate places with a frequency of at least twice a week for at least 3 consecutive months in children older than 5 years of age, or encopresis, which is an involuntary or intentional repeated passage of feces into inappropriate places. At least one such event occurs each month for at least 3 months for children older than 4 years [1]. The occurrence is not attributed to any underlying anatomic or neurologic abnormalities and cannot be the direct effect of a substance’s [2].

Elimination disorder has been found to be prevalent worldwide; it affects around 0.7 percent to 29.6 percent of the paediatric population. Daytime urinary incontinence at age 7 varies from 6.3 to 9.0%; about 10% to 20% of 7–year-olds get their beds wet on a regular basis; and the magnitude of encopresis among the paediatric population ranges from 0.3 to 8% of children in Western society [3–6].

Elimination disorder leads to social, family, and psychological problems. The problem impacts the lives of children and adolescents and puts them at risk for social isolation, peer conflict, teasing, and classroom challenges. As a result, children and adolescents with elimination disorders often suffer from low self-esteem and psychological problems. But in so many other areas of human life, the scientific approach to human waste elimination disorders has dramatically reduced the meanings attributed to them and almost all of the problems they can cause [7, 8].

Parents and teachers are poorly informed about barriers to paediatric elimination disorder; as a result, its magnitude is under reported, and children and adolescents with elimination disorder are less understood by medical and mental health professionals [9, 10]. Despite its serious effects on children, adolescents, families, and society, the magnitude of elimination disorder is poorly understood due to limited research on it [8]. Furthermore, the prevalence of enuresis and encopresis is rarely studied in developing countries, despite the fact that there are factors in these countries that could affect it [11]. Studying the prevalence and associated factors of elimination disorder has critical input to good physical function and outcomes in children and adolescents [7]. Moreover, up to the level of the researchers search and knowledge, there is a lack of adequate information about elimination disorders among the paediatric population in Ethiopia. So, the current study aimed to assess elimination disorder and its associated factors among children and adolescents aged 5–14 years old attending the paediatric outpatient Clinic at Wolaita Sodo University Comprehensive Specialized Hospital in Wolaita Sodo, South Ethiopia.

## Methods and materials

### Study area and period

A study was conducted at Wolaita Sodo University Comprehensive Specialized Hospital (WSUCSH) from September 22 to November 22, 2022. The hospital is located in Wolaita Sodo town, 329 km south of Addis Ababa, the capital city. The hospital delivers different medical services for outpatients, emergency patients, and inpatients for approximately 450–500 patients per day, and the total service coverage of the hospital is about more than 3 million people in its catchment areas. The paediatric department has six major wings: paediatric outpatient, paediatric Emergency (admission and outpatient unit), Neonatal Intensive Care Unit, paediatric surgical admission ward, paediatric medical admission ward, and stabilization center unit.

### Study design

A hospital-based cross-sectional study design was conducted.

### Population

All children and adolescents ages 5–14 who attended WSUCSH Paediatric outpatient department services during the data collection period were included in the study. Children and adolescents who were critically ill to the extent of being unable to communicate during data collection and who had known anatomical abnormalities of the urinary tract or bowel due to medical (biogenic or neurological) causes were excluded from the study.

### Sampling technique and procedure

The required sample size for this study was determined by using a single population proportion formula with the following assumption: estimated prevalence (P) of elimination disorder at 50%, as there is a lack of published studies that show the magnitude and associated factors of elimination disorder in Ethiopia, a 95% confidence interval (CI), a 5% margin of error (W), and a 10% non-response rate. Accordingly, the final sample size was 423. A systematic random sampling technique was used to select the study participants. To select the desired sample, the average number of paediatric patients who visited the paediatric outpatient department within the last three months before the study was identified from the client registration. On the basis of this, the expected client flow rate during the study period was estimated to be 2350. Then the sampling interval (k) was calculated by dividing the expected number of patients visiting the unit during the study period (N) by the determined sample size (n) of respondents, and it was found to be 5. The lottery method was used to select the first subject from interval 1−K, and then every K interval of the sample was selected up to collect the required sample size.

### Study variable

The dependent variable was Elimination disorder, and the independent variables were socio-demographic characteristics (child sex, child age, ethnicity, religion, residence, child educational level, family marital status, and parent’s educational level, parental occupation, living circumstances of the child, family size, and family average monthly income). Clinical and biological factors of children and their mothers (maternal substance use, family history of elimination disorder, terms of pregnancy, route of delivery, duration of labor, snoring, and child exclusive breastfeeding method during the first 6 months), Psychosocial factors (history of post-traumatic or stressful events, child’s difficult behavior (behavioral problems, hyperactivity, conduct, and emotional problems), parenting practices [good (positive) parenting and bad parenting (inconsistent discipline and poor supervision), toilet training skill (low, medium, and high), and method to assist child’s elimination problem (Punishment as discipline and giving sugary beverage)].

### Data collection instrument

#### Development of Symptom Score for Dysfunctional Elimination Syndrome (DSSDES)

The presence of elimination disorder among children and adolescents aged 5–14 years old was assessed using a new valid Development of Symptom Score for Dysfunctional Elimination Syndrome (DSSDES) tool. The questionnaire contained two measures in which Vancouver/DSS/ and DES questioners had a 14-item condition-specific measure to evaluate symptoms of bladder or enuresis (items 1–10) and bowel dysfunction or encopresis (items 11–13). The last item, number 14, which evaluates the difficulty of the measurement but is not used to assess elimination disorder since it evaluates how easy it was to answer the item number 1–13 questions, for this reason, the tool recommends excluding it during scoring, and all remaining items are weighted equally. All item responses are scored using a 5-point Likert scale, with scores ranging from 0 (no complaints) to 4 (severe symptoms). Total scores range from 0 to 52, with a cutoff score of ≥ 11 for the DSSDES of Vancouver questioners with a sensitivity of 80% and a specificity of 91% have the ability to detect paediatric elimination disorders [12]. The presence of enuresis was assessed by a cutoff score ≥ 8.5 for items 1 to 10 in the DSSDES of Vancouver questioners, which is adopted and validated from the dysfunctional voiding symptom score (DVISS) [13] and encopresis was assessed by using a cutoff score ≥ 3.5 for items 11 to 13 in the DSSDES of Vancouver questioners, which is adopted and validated from the parental questionnaire on enuresis and urinary incontinence, PQ_EnU [14]. The presence of nocturnal and diurnal enuresis is assessed by the DSM-5 definition as wetting at night or during the day with a frequency of at least twice a week for at least three consecutive months [15]. The DSSDES Rating Scale was pretested for reliability in the current study setting and was found to be easily understood by the participants with internal consistency (Cronbach’s alpha = 0.86).

### Strengths and Difficulties Questionnaire Parent Report (SDQ-PR)

Children and adolescents with difficult behavioral problems were assessed by the strengths and difficulties questionnaire parent report (SDQ-PR). It has 25 items subdivided into five subscales of five items each, which measure hyperactivity, emotional symptoms, conduct problem symptoms, interpersonal relationships, and pro-social behavior. A 25-item 3-point Likert scale with a total score of 0–40 without a pro-social behavioral subscale ‘Somewhat true’ is always scored as 1, but the scoring of not True’ and certainly True’ varies with the item. Without the pro-social behavioral subscale, the overall optimum cutoff point ≥ 17 of the SDQ-PR has the ability to screen behavioral problems with sensitivity of 70.96% and specificity of 69.15%, and the optimum cutoff score for subscales is ≥ 7 for hyperactive-inattentive problems, ≥ 4 for conduct, and ≥ 5 for emotional problems [16]. The SDQ-PR rating scale was pretested for reliability in our setup and was found to have internal consistency (Cronbach’s alpha = 0.79).

### Child Trauma Screening Questionnaire (CTSQ)

Children's and adolescents trauma was assessed by the CTSQ. The CTSQ assesses re-experiencing (5 items) and hyper-arousal symptoms (5 items). The response was yes (scored 1) or no (scored 0) to whether they have experienced the symptoms since the event, and an optimal cutoff score of ≥ 5 was derived as providing the best prediction of whether children and adolescents have trauma [17]. In the current study setting the internal consistency of Cronbach’s alpha for the scale was 0.76.

### Alabama Parenting Questionnaire APQ-9

Child and adolescent parenting practices were assessed by the APQ-9. It has three items chosen for each of the factors of good parenting (positive) and bad parenting (inconsistent discipline and poor supervision). APQ-9 item 5-point Likert scale: never (1), almost never (2), sometimes (3), often (4), always (5). Mean scores of 4.48 and above indicate good parenting and bad parenting (mean scores of 2.73 to 4.48 indicate inconsistent discipline, and mean scores of 1 to 2.73 indicate poor supervision) [18]. In our setup, the internal consistency of Cronbach’s alpha was 0.871.

### Paediatric-assessment-tool/toilet training/-for-issuing-of-products

Toilet training skills for children and adolescents were assessed by the paediatric assessment tool for toilet training skills for the issuing of products. The tool has 11 items with different Likert scales, which are scored as follows: score ≥ 30 has low toilet training skill; score 17–29 has medium toilet training skill; score ≤ 16 has high toilet training skill [19]. The internal consistency of Cronbach’s alpha in the current study was 0.799. Other questionnaires adopted from previous studies for possible associated factors related to elimination disorder were methods to assist children with elimination disorder, socio-demographic variables, and biological or clinical factors included [20, 21].

### Data collection procedure

Data were collected from parents for children aged 5 to 8 years, and from children and adolescents aged 9 to 14 years, a structured face-to-face interviewer-administered questionnaire was used. Data were collected by six trained Bachelor of Science degree holders in psychiatry and supervised by two Mental Health specialists.

### Data quality management

The questionnaire was first prepared in English, translated into Amharic and Wolaita, and then re-translated into English by experts in all three languages, including mental health specialists, to check its consistency. Two days of training were given for data collectors and supervisors. Reliability of tools was checked, and a pre-test was conducted for 5% (n = 22) of the sample size at Humbbo Tebela primary hospital, 20 km away from the study area, to identify potential problems in data collection tools and modification of the questionnaires. Regular supervision and support were given to data collectors by the supervisors and principal investigator. Data was checked for completeness and consistency by supervisors and principal investigators on a daily basis during data collection time.

### Data processing, analysis and presentation

Data were entered into Epi Data Version 4.6 and analyzed using SPSS version 25 statistical software; descriptive statistics were used to describe the sample characteristics and assess the magnitude of ED. Multicollinearity was checked by the variance inflation factor (VIF < 2), which indicates that independent variables are not correlated to each other or there is no Multicollinearity and the selected model was a good logistic regression model fit, since the Hosmer–Lemeshow goodness-of-fit P-value was 0.58 it is greater than 0.05. The association between independent variables and the outcome variable was investigated using logistic regression analysis. Variables with a p-value < 0.25 in bivariate binary logistic regression analysis were entered into multivariable logistic regression analysis and variables with a p-value < 0.05 in multivariable logistic regression analysis were considered to have a significant association. A 95% CI and Odds ratio with corresponding p-value < 0.05 were used to determine the predictors of the outcome variable.

## Result

### Socio-demographic characteristics of participants

A total of 423 children and adolescents were invited to participate in the study, and 417 completed the interview with a response rate of 98.6%. Out of the total participants (n 271, 65%) were males, and the educational level of children and adolescents (n 270, 64.7%) was primary school and above. The mean age of participants was 8.3 years, with a standard deviation of (SD + 2.31). Regarding the living circumstances of kids (n 297, 71.2%), they were living with their parents, and half of participants (n 214, 51.3%) live in a family size of less than four (Table 1).

**Table 1:** Socio-demographic and family related characteristics of children and adolescents age 5–14 year old attending paediatric outpatient, at Wolaita sodo university comprehensive specialized hospital, Wolaita sodo, south Ethiopia, 2022

Variable	Category	Frequency	Percentage (%)
Age	5–8 year old	247	59.2
	9–11 year old	113	27.1
	12–14 year old	57	13.7
Child sex	Male	271	65.0
	Female	146	35.0
Residency	Urban	241	57.8
	Rural	176	42.2
Religion	Orthodox	124	29.7
	Muslim	64	15.3
	Protestant	185	44.4
	Others^a^	44	10.6
Ethnicity	Wolaita	287	68.8
	Amhara	56	13.4
	Gurage	38	9.1
	Oromo	13	3.1
	Others^b^	23	5.5
Educational level of child	Kindergarten (KG)	147	35.3
	Primary and above	270	64.7
Currently living	With parents	297	71.2
	Steep parents	62	14.9
	Residential institution	30	7.2
	Gordian	28	6.7
Family size	< 4	214	51.3
	≥ 4	203	48.7
Occupation of parents	Government employ	144	34.5
	Private	54	12.9
	Merchant	64	15.3
	Farmer	62	14.9
	Housewife	19	4.6
	Unemployed	35	8.4
	Daily labor	39	9.4
Educational status of parents	Illiterate	84	20.1
	Primary school	182	43.6
	High school and above	151	36.2
Parents marital status	Married	298	71.5
	Divorced	48	11.5
	Separated	37	8.9
	Widowed	23	5.5
	Single	11	2.6
Average family monthly income	< 1000	61	14.6
	1000–2500	60	14.4
	2500–3400	70	16.8
	≥ 3400	226	54.2

^a^Others religion (7th day Adventist, Catholic)

^b^Ethnicity (Gamo, Konso, Hadiya, Kambata, Maraqo)

### Clinical or biological factors of participants

Nearly three-quarters of the study participants (n 315, 75.5%) had a full-term pregnancy, and greater than half of participants (n 243, 58.3%) had duration of labor less than ten hours. In this study (n 122, 29.3%), participants had a family history of elimination disorder (Table 2).

**Table 2:** Clinical or biological factors of children and adolescents aged 5–14 year old attending paediatric outpatient in Wolaita sodo university comprehensive specialized hospital, Wolaita sodo, south Ethiopia, 2022

Variable	Category	Frequency	Percentage (%)
Period of gestation	Full term	315	75.5
	Pre-term	75	18.0
	Post term	27	6.5
Duration of labour	< 10 h	243	58.3
	≥ 10 h	174	41.7
Mode of delivery	Normal vaginal	311	74.6
	Vacuum delivery	60	14.4
	Cesarean-section	46	11.0
Child exclusive breastfeeding method first six month	Bottle only	90	21.6
	Breast and bottle	327	78.4
Maternal substance use	Yes	110	26.4
	No	307	73.6
Alcohol	Yes	43	10.3
	No	374	89.7
Cigarette	Yes	13	3.1
	No	404	96.9
Khat	Yes	32	7.7
	No	385	92.3
Others^a^	Yes	26	6.2
	No	391	93.8
Family history of elimination disorder	Yes	122	29.3
	No	295	70.7
Does child have snoring	Yes	188	45.1
	No	229	54.9

^a^Others substance (shesha, cannabis, opioids)

### Psychosocial related factors of participants

Just over a third of study participants (n 154, 36.9%) were exposed to traumatic life events, and among children and adolescents who were exposed to traumatic situations (n 73, 17.5%), they had post-traumatic stress problems. Participants parents think that out of 417 participants, children and adolescents (n 172, 41.2%) have elimination problems, and in order to improve their elimination problems, parents have tried limiting food and fluids (n 62, 14.9%), washing their own clothes as punishment (n 50, 12%), and giving sugary beverages as rewards (n 99, 23.7%). Among the participants, children and adolescents (n 137, 32.9%) had emotional problems and (n 73, 17.5%) had hyperactive problems. Regarding parenting practices (n 42, 10.1%), there were good (positive) parenting practices and bad parenting practices were (n 113, 27.1%) inconsistent discipline, and (n 262, 62.8%) poor supervision. Out of the total participants, children and adolescents (n 262, 62.8%) had high toilet training skills; (n 88, 21.1%), had medium toilet training skills; and (n 67, 16.1%) had low toilet training skills (Table 3).

**Table 3:** Psychosocial characteristics of respondents and their children and adolescents age 5–14 year old attending paediatric outpatient, at Wolaita sodo university comprehensive specialized hospital, Wolaita sodo, south, Ethiopia 2022

Variable	Category	Frequency	Percentage (%)
Do you have experienced accidents or stress full life events any?	Yes	154	36.9
	No	263	63.1
Child post-traumatic stress problem	Yes	73	17.5
	No	344	82.5
Child behavioral problem	Yes	117	28.1
	No	300	71.9
Child emotional problem	Yes	137	32.9
	No	280	67.1
Child conduct problem	Yes	89	21.3
	No	328	78.7
Child hyperactive problem	Yes	73	17.5
	No	344	82.5
Parenting practices of child	Positive parenting	42	10.1
	Inconsistent-discipline	113	27.1
	Poor supervision	262	62.8
Do you think your child had elimination problem?	Yes	172	41.2
	No	245	58.8
Clap and belt child to punishment	Yes	61	14.6
	No	356	85.4
Restrict food and fluid to assist	Yes	62	14.9
	No	355	85.1
Wash own cloth to punishment	Yes	50	12
	No	367	88
Giving sugary beverages to assist child elimination problem	Yes	99	23.7
	No	318	76.3
Child toilet training skill	High	262	62.8
	Medium	88	21.1
	Low	67	16.1

### Magnitude of elimination disorder

**Fig. 1:**
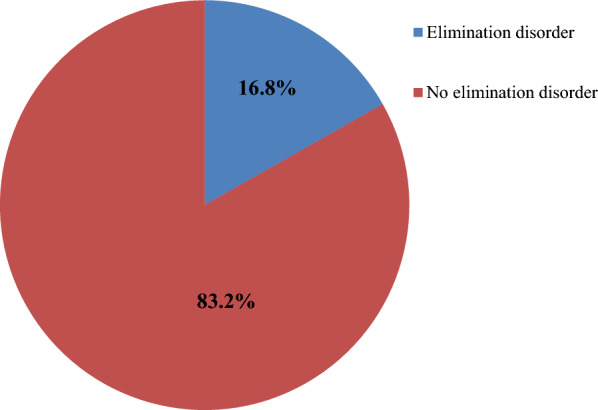
Magnitude of elimination disorder among children and adolescents age 5–14 year old attending paediatric outpatient at Wolaita Sodo university comprehensive specialized hospital, Wolaita sodo, south Ethiopia 2022

Magnitude of overall elimination disorder among children and adolescents age 5–14 year old was (n 70, 16.8%) with 95%CI [13.3, 20.7] (Fig. 1).

### Description of elimination disorder domains

**Fig. 2:**
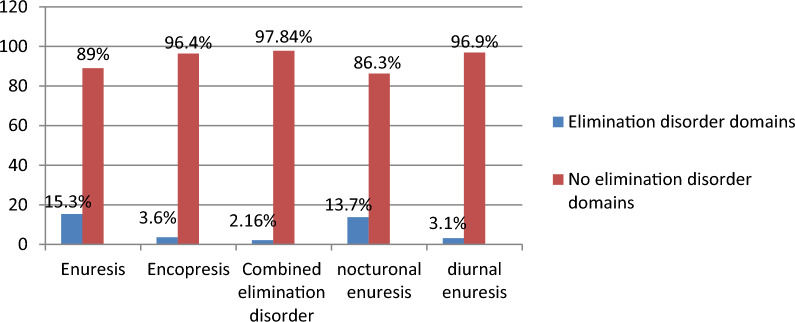
Description of elimination disorder domains among children and adolescents age 5–14 year old attending paediatric outpatient at Wolaita Sodo university comprehensive specialized hospital, Wolaita sodo, south Ethiopia 2022

Elimination disorder has two domains, which are enuresis and encopresis. Overall prevalence of enuresis was (n 64, 15.3%) and encopresis (n 15, 3.6%), both enuresis and encopresis or combined elimination disorder (n 9, 2.2%) among children and adolescents (Fig. 2).

### Description of elimination disorder by sex and age of participants

**Fig. 3:**
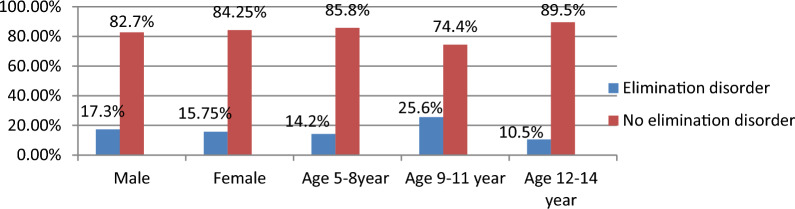
Description of elimination disorder by age and sex among children and adolescents age 5–14 year old attending paediatric outpatient at Wolaita Sodo university comprehensive specialized hospital, Wolaita Sodo, south Ethiopia 2022

In this study, out of 417 participants, the total magnitude of elimination disorder was higher in boys (n 47, 17.3%) and girls (n 23, 15.75%). Similarly (n 35, 14.2%) in age 5–8 year old, (n 29, 25.6%) in age 9–11 year old, and (n 6, 10.5%) in 12–14 year old children and adolescents (Fig. 3).

### Description of Nocturnal and Diurnal enuresis by age of participants

**Fig. 4:**
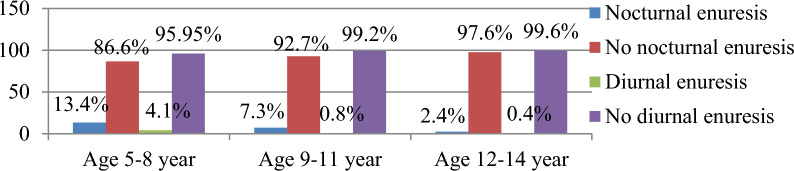
Description of nocturnal and diurnal enuresis by age among children and adolescents age 5–14 year old attending paediatric outpatient at Wolaita Sodo university comprehensive specialized hospital, Wolaita Sodo, south Ethiopia 2022

In this study, Enuresis was the most common elimination disorder; nocturnal enuresis was higher than diurnal enuresis, and its magnitude decreased as age increased. In this study, the total prevalence of Nocturnal enuresis was (n 57, 13.7%); [Nocturnal enuresis in age 5–8 years (n 33, 13.4%), 9–11 years (n 18, 7.3%), and 12–14 years (n 6,2.4%)]. and Diurnal enuresis (n 13, 3.1%); [Diurnal enuresis in age 5–8 years (n 10, 4.05%), 9–11 years (n 2, 0.8%), and 12–14 years (n 1, 0.4%)] (Fig. 4).

### Factors associated with elimination disorder

Table 4 represents bivariable analysis of socio-demographic and family related factors and Table 5 represents bivariable analysis of clinical, psychosocial and family related factors. In the current study younger age 9–11 year old, children and adolescents living in family size of four and above, family history of elimination disorder, children and adolescents had emotional and hyperactive problem, bad parenting practices were poor supervision, low toilet training skill were significantly associated with elimination disorder (Table 6).

**Table 4:** Bivariable analysis of socio-demographic and family related factors of children and adolescents aged 5–14 year old attending paediatric outpatient at Wolaita sodo university comprehensive specialized hospital, Wolaita sodo, south Ethiopia 2022

Variable	Category	Elimination disorder		COR (95% CI)	P-value
		Yes n (%)	No n (%)		
Age of child	5–8 year	35 (14.2%)	212 (85.8%)	1.4 (0.56, 3.52)	0.47
	9–11 year	29 (25.6%)	84 (74.4%)	2.9 (1.14, 7.55)	0.026*
	12–14 year	6 (10.5%)	51 (89.5%)	1	1
Child sex	Male	47 (17.3%)	224 (82.7%)	1.1 (0.65, 1.93)	0.68
	Female	23 (15.75%)	123 (84.25%)	1	1
Residency	Urban	29 (12%)	212 (88%)	1	1
	Rural	41 (23.3%)	135 (76.7%)	2.2 (1.32, 3.74)	0.003*
Educational level of child	Kindergarten (KG)	29 (19.7%)	118 (80.3%)	1.4 (0.81, 2.32)	0.23*
	Primary and above	41 (15.2%)	229 (84.8%)	1	1
Currently living	With parents	49 (16.5%)	248 (83.5%)	1	1
	Steep parents	8 (12.9%)	54 (87.1%)	0.75 (0.34, 1.67)	0.48
	Residential institution	6 (20%)	24 (80%)	1.3 (0.49, 3.26)	0.63
	Guardian	7 (25%)	21 (75%)	1.7 (0.68, 4.18)	0.26
Family size	< 4	25 (11.7%)	189 (88.3%)	1	1
	≥ 4	45 (22.2%)	158 (77.8%)	2.2 (1.26, 3.67)	0.005*
Occupation of parents	Government	28 (19.4%)	116 (80.4%)	1	1
	Private	9 (16.7%)	45 (83.3%)	0.83 (0.36, 1.89)	0.65
	Merchant	12 (18.75%)	52 (81.25%)	0.96 (0.45, 2.03)	0.9
	Farmer	6 (9.67%)	56 (90.3%)	0.4 (0.17, 1.13)	0.09*
	Housewife	5 (26.3%)	14 (73.7%)	1.4 (0.49, 4.45)	0.48
	Unemployed	5 (14.3%)	30 (85.7%)	0.69 (0.25, 1.94)	0.48
	Daily labor	5 (12.8%)	34 (87.2%)	0.6 (0.22, 1.69)	0.34
Educational level of parents	Illiterate	13 (15.5%)	71 (84.5%)	0.88 (0.43, 1.82)	0.73
	Primary school	31 (17%)	151 (83%)	0.98 (0.56, 1.75)	0.96
	High school and above	26 (17.2%)	125 (82.8%)	1	1
Parental marital status	Married	48 (16%)	250 (84%)	1	1
	Divorced	5 (10.4%)	43 (89.6%)	0.61 (0.23, 1.61)	0.3
	Separated	7 (18.9%)	30 (81.1%)	1.2 (0.51, 2.93)	0.7
	Widowed	5 (21.7%)	18 (78.3%)	1.4 (0.51, 4.08)	0.49
	Single	5 (45.5%)	6 (54.5%)	4.3 (1.27, 14.8)	0.019*
Average family income	< 1000	6 (9.84%)	55 (90.16%)	0.507 (0.204,1.26)	0.14*
	1000–2500	8 (13.3%)	52 (86.7%)	0.72 (0.32, 1.62)	0.42
	2500–3400	16 (22.85%)	54 (77.15%)	1.37 (0.72, 2.65)	0.34
	≥ 3400	40 (17.7%)	186 (82.3%)	1	1

^*^Factors that have association at p-value < 0.25 1 = reference category

**Table 5:** Bivariable analysis of clinical, psychosocial and family related factors of children and adolescents aged 5–14 year old attending paediatric outpatient at Wolaita sodo university comprehensive specialized hospital, Wolaita sodo, south Ethiopia 2022

Variable	Category	Elimination disorder		COR (95% CI)	P-Value
		Yes n (%)	No n (%)		
Gestational period	Full term	51 (16.2%)	264 (83.8%)	1	1
	Pre-term	14 (18.7%)	61 (81.3%)	1.18 (0.62, 2.28)	0.61
	Post-term	5 (18.5%)	22 (81.5%)	1.17 (0.43, 3.25)	0.75
Duration of labour	< 10 h	39 (16.1%)	204 (83.9%)	1	1
	≥ 10 h	31 (17.8%)	143 (82.2%)	1.13 (0.67, 1.90)	0.63
Mode of delivery	Normal vaginal	53 (17%)	258 (83%)	1	1
	Vacuum	10 (16.7%)	50 (83.3%)	0.97 (0.46, 2.04)	0.94
	Cesarean-section	7 (15.2%)	39 (84.8%)	0.87 (0.37, 2.06)	0.76
Exclusive breastfeeding method 1st 6 month	Only bottle feeding	16 (17.7%)	74 (82.3%)	1.09 (0.59, 2.02)	0.78
	Bottle and Breast-feeding	54 (16.5%)	273 (83.5%)	1	1
Maternal Substance use	Yes	19 (17.3%)	91 (82.7%)	1.05 (0.59, 1.87)	0.87
	No	51 (16.6%)	256 (83.4%)	1	1
Alcohol	Yes	6 (13.9%)	37 (86.1%)	0.78 (0.32, 1.94	0.6
	No	64 (17.1%)	310 (82.9%)	1	1
Cigarette	Yes	2 (15.4%)	11 (84.6)	0.89 (0.19, 4.14	0.9
	No	68 (16.8%)	336 (83.2%)	1	1
Khat	Yes	7 (21.8%)	25 (78.2)	1.4 (0.59, 3.45)	0.43
	No	63 (16.4%)	322 (83.6%)	1	1
Others	Yes	6 (23%)	20 (77%)	1.5 (0.59, 3.96)	0.38
	No	64 (16.4%)	327 (83.6%)		
Family history of elimination disorder	Yes	38 (31%)	84 (69%)	3.72 (2.19, 6.32)	< 0.001*
	No	32 (10.8%)	263 (89.2%)	1	1
Your child have snoring	Yes	34 (18.1%)	154 (81.9%)	1.18 (0.71, 1.98)	0.52
	No	36 (15.7%)	193 (84.3%)	1	1
Post-traumatic stress disorder	Yes	14 (19.2%)	59 (80.8%)	1.2 (0.64, 2.34)	0.54
	No	56 (16.3%)	288 (83, 7%)	1	1
Behavioral problem	Yes	25 (21.4%)	92 (78.6%)	1.54 (0.89, 2.65)	0.12*
	No	45 (15%)	255 (85%)	1	1
Emotional problem	Yes	36 (26.3%)	101 (73.7%)	2.57 (1.53, 4.35)	< 0.001*
	No	34 (12%)	246 (88%)	1	1
Conduct problem	Yes	13 (14.6%)	76 (85.4%)	0.81 (0.42, 1.56)	0.54
	No	57 (17.4%)	271 (82.6%)	1	1
Hyperactive problem	Yes	24 (32.9%)	49 (67.1%)	3.17 (1.78, 5.66)	< 0.001*
	No	46 (13.4%)	298 (86.6%)	1	1
Parenting practices of child	Positive	5 (11.9%)	37 (88.1%)	1	1
	Inconsistent discipline	8 (7.1%)	105 (92.9%)	0.56 (0.17, 1.83)	0.34
	Poor supervision	57 (21.7%)	205 (78.3%)	2.1 (0.77, 5.47)	0.15*
Clap and belt the child	Yes	13 (21.3%)	48 (78.7%)	1.4 (0.72, 2.79)	0.31
	No	57 (16%)	299 (84%)	1	1
Restrict food and fluid	Yes	12 (19.3%)	50 (80.7%)	1.2 (0.62, 2.45)	0.56
	No	58 (16.3%)	297 (83.7%)	1	1
Wash own cloth	Yes	11 (22%)	39 (78%)	1.5 (0.71, 3.04)	0.3
	No	59 (16%)	308 (84%)	1	1
Giving sugary beverage	Yes	20 (20.2%)	79 (79.8%)	1.3 (0.76, 2.41)	0.3
	No	50 (15.7%)	268 (84.3%)	1	1
Child toilet training skill	High	41 (15.6%)	221 (84.4%)	1	1
	Medium	10 (11.4%)	78 (88.6%)	0.69 (0.33, 1.44)	0.33
	Low	19 (28.4%)	48 (71.6%)	2.1 (1.14, 3.99)	0.018*

^*^Factors that have association at p-value < 0.25 1 = reference category

**Table 6:** Multivariable analysis of factors associated with Elimination disorder among children and adolescents age 5–14 year old attending paediatric outpatient at Wolaita sodo university comprehensive specialized hospital, Wolaita sodo, south Ethiopia 2022

Variable	Category	Elimination disorder		COR(95%CI)	AOR (95% CI)	P-value
		Yes n (%)	No n (%)			
Age	5–8 year	35 (14.2%)	212 (85.8%)	1.4 (0.56, 3.52)	1.06 (0.38, 2.98)	0.91
	9-11 year	29 (25.6%)	84 (74.4%)	2.9 (1.14, 7.55)	3.2 (1.09, 9.43)	0.03*
	12–14 year	6 (10.5%)	51 (89.5%)	1	1	1
Family size	< 4	25 (11.7%)	189 (88.3%)	1	1	1
	≥ 4	45 (22.2%)	158 (77.8%)	2.2 (1.26, 3.67)	3.4 (1.78, 6.56)	< 0.001*
Family history of elimination disorder	Yes	38 (31%)	84 (69%)	3.7 (2.19, 6.32)	3.9 (2.12, 7.45)	< 0.001*
	No	32 (10.8%)	263 (89.2%)	1	1	1
Emotional problem	Yes	36 (26.3%)	101 (73.7%)	2.6 (1.53, 4.35)	2.2 (1.18, 4.05)	0.013*
	No	34 (12%)	246 (88%)	1	1	1
Hyperactive problem	Yes	24 (32.9%)	49 (67.1%)	3.2 (1.78, 5.66)	3.8 (1.83, 7.83)	< 0.001*
	No	46 (13.4%)	298 (86.6%)	1	1	1
Child parenting practice	Positive	5 (11.9%)	37 (88.1%)	1	1	1
	Inconsistent discipline	8 (7.1%)	105 (92.9%)	0.6 (0.17, 1.83)	0.93 (0.23, 3.70)	0.92
	Poor supervision	57 (21.7%)	205 (78.3%)	2.1 (0.77, 5.47)	4.4 (1.29, 14.69)	0.018*
Child toilet training skill	High	41 (15.6%)	221 (84.4%)	1	1	1
	Medium	10 (11.4%)	78 (88.6%)	0.7 (0.33, 1.44)	1.2 (0.49, 2.78)	0.73
	Low	19 (28.4%)	48 (71.6%)	2.1 (1.14, 3.99)	5.9 (2.61, 13.33)	< 0.001*

^*^Factors that have association at p-value < 0.05 on multivariable analysis, 1 = reference category

## Discussion

In this study, the total magnitude of elimination disorders was 16.8%, which is consistent with a similar study conducted in Germany (14.8%), Australia (18.2%), Egypt (15.7%) and Kenya (14.5%) [22–25]. However, the magnitude of elimination disorder in this study is higher than the studies conducted in Iran (5.4%), the United States (4.45%), and Hong Kong (3.1%) [26–28]. The possible reason for the difference between the Iran study and the current study is that the former was done in the community among children age 6–18 years and used the Kiddie-Schedule for Affective Disorders and Schizophrenia for School-Age Children—Present and Lifetime Version (K-SADS-PL DSM-IV) to discriminate elimination disorders, whereas the present study is hospital-based among children and adolescents aged 5–14 years and used the DSSDES tool and DSM-5 to identify elimination disorders. Furthermore, differences between the United States study and the present study might be explained by differences in the data collection tools employed, as they used a computerized version of the Diagnostic Interview Schedule for Children (C-DISC 4.0) to detect elimination disorder, and their ages ranged from 8 to 11 years. Furthermore, the Hong Kong study differed from this one in that it was conducted in a school setting and used only symptoms criteria to differentiate elimination disorders, such as frequency of wetting or soling. However, this study applied standard instruments, the DSSDES tool and the DSM-5, to detect elimination disorder.

The magnitude of elimination disorder found in this study is lower than studies conducted in southern Brazil (35.2%), China (59%), and Korea (46.4%), covering both enuresis and encopresis [29–31]. The difference between the study conducted in southern Brazil and the present study might be that the former was performed by using DVISS to measure elimination disorder among 580 samples of children in an urban community; however, the present study is conducted in a hospital setting among 417 samples and uses different tools to identify elimination disorder. Additionally, the difference between China and our study could be that the former was performed by using pediatric dysfunctional voiding scales to measure the frequency of enuresis or encopresis to identify elimination disorders in 156 samples from 10 different countries, whereas the present study collected 417 participants from a single study area. Another possible difference between the Korea and current studies is that the former involved 19,240 children (5–13 years old), and elimination disorder was measured using dysfunctional voiding symptoms (DVSs) and abnormal bowel habits (ABHs). Whereas the current study is conducted in 417 children (5–14 years old) and DSM-5 criteria are used to identify elimination disorder in addition to the Korea study.

In this study, younger age groups (9–11 years) were 3.2 times more likely to have elimination disorder than older age groups (12–14 years), which is in line with studies conducted in Turkey, Iraq, and Santo Domingo, Dominican Republic [32–34]. For a number of reasons, an association between elimination disorder and age has been described. The most crucial reason was that as age increased, elimination disorders decreased. Furthermore, it appeared to be an elimination disorder related to age, psychological development, and physical development to achieve bladder and bowel control at the expected age [7, 35]. According to evidence, elimination disorder drops by 20% in 5-year-old children and by 1–2% by the end of adolescence. The prevalence of enuresis was similarly found in this study: nocturnal enuresis in ages 5–8 was 13.4%, 9–11 was 7.3%, 12–14 was 2.4%, and diurnal enuresis in ages 5–8 was 4.05%, 9–11 was 0.8%, and 12–14 was 0.4%, with a mean age of 8.3 years. This is consistent with the study mentioned in the Synopsis of Psychiatry book, which reported that by age 7 years the prevalence was reported to be 15.2%, by age 10 the overall prevalence was reported to be 3%, and the rate drops dramatically for teenagers aged 14 years, where the prevalence is only 1.5 percent [36]. Another possible explanation might be that children get better at understanding problems as they get older. However, younger kids have a harder time understanding how to rationally solve problems than older kids, which makes them less aware of elimination disorders and more likely to accept inappropriate urination or defecation as a solution to their problem rather than reporting their parents to obtain medical care [37].

Similarly, the odds of elimination disorder among children and adolescents living in family sizes of four and above were 3.4 times higher than the odds in children and adolescents who reside in family sizes below four, which is in agreement with a study done in Turkish [38] who discovered that the odds of elimination disorder were higher in children from large families than small families. This could be due to a lack of family support for the child or a child’s refusal to use the bathroom to satisfy an unsatisfied psychological need due to family size, which exposes them to elimination disorders. Elimination disorder was also more common in larger families; the main reason may be the stress associated with jealousy and anxiety that exists in the family, where attention is diverted toward other relatives who live in the house [32, 39]. Furthermore, in large families, most family problems usually arise when there is a lot of conflict or tension for different reasons. In such families, children do not feel safe or secure. Such children tend to internalize their feelings and bottle them deep inside. It can lead to elimination disorders as they try to find a way to express themselves and release their suppressed emotions [40].

In this study, children and adolescents who have a family history of elimination disorder were 3.9 times more likely to have this disorder than those who have no family history of elimination disorder; this was also reported in previous studies conducted in Taiwan which explained that genetically parents who have this disorder increase the occurrence of this disorder in kids. Furthermore regions on chromosomes 8, 12, and 13 are associated with a higher risk of elimination disorder in children and adolescents [41].

According to the findings of this study, the odds of having an elimination disorder were 2.2 times higher among children and adolescents who have emotional problems compared to those who do not. This finding was supported by previous studies conducted in Belgium and Iran [42, 43]. This association might be linked to elimination disorder because it can lead to embarrassment for the child or adolescents and disappointment for parents. Children and adolescents with emotional problems can have behavior problems that interfere with toilet training and refuse to use the bathroom, so children and adolescents with ED are at higher risk for emotional problems [42–45]. Nevertheless, elimination disorder in children and adolescents with psychological disorders like emotional problems is the most common, and vice versa [46, 47].

This study observed that the odds of having elimination disorder were 3.8 times higher among children and adolescents who have hyperactive problems compared to those who have no hyperactive problems. This is in agreement with a study conducted in Germany [22]. One possible explanation for this association is that children and adolescents who have a hyperactive problem accept elimination disorder as a normal occurrence, and they sometimes have a reluctance to use the toilet room due to a preoccupation with play activities that may aggravate elimination symptoms or put them at high risk of developing elimination disorder [48, 49].

According to this study, the odds of having an elimination disorder were 4.4 times higher in children and adolescents who had bad parenting practices (poor supervision) than in those who had good (positive) parenting practices. This finding was similar to previous studies conducted in China and South Africa [50, 51]. One possible reason might be that children and adolescents who have bad parenting practices (poor supervision) can experience parental corporal punishment in the form of hitting, punching, kicking, or beating. This can cause kids to be preoccupied with anxiety or fear in response to their parents’ poor parenting practices; as a result, the children and adolescents may have nightmares reliving terrifying trauma, which causes them to wake up suddenly and urinate or defecate in their bed or underwear. Furthermore, this makes them reluctant to report their elimination problem to their parents, putting them at high risk of developing an elimination disorder [38, 50–52].

This study observed a significant association between elimination disorder and low toilet training skill; children and adolescents with low toilet training skill were 5.9 times more likely to have an elimination disorder than those with high toilet training skill. This finding was the same as that of a study conducted in Nigeria [53]. A possible explanation for this association might be that starting toilet training skills without regard for the child’s emotional readiness or cooperation makes the child reluctant to learn toilet training methods, resulting in low toilet training skills, which puts the child and adolescents at high risk for elimination disorder [54, 55]. Also, maybe there was an incorrect toilet training method or child’s behavioral problems that can cause the child or adolescents to attain low toilet training skill, which leads to significant physical and psychological consequences and persistent elimination symptoms, such as enuresis and encopresis [56, 57]. A child or adolescents will develop a sense of autonomy that will eventually lead them to the virtue of wellbeing if they are successfully skilled in toilet training within the appropriate years. Yet if the children and adolescents are unable to do so, it may result in a psychological crisis of shame and doubt. These crises frequently cause embarrassment among peers and have been linked to psychological and elimination disorders in kids [58, 59].

## Limitation of the study

This study is not without limitations; the use of non-standardized questions to measure mental health care service delivery readiness may undermine confidence in the findings. The study utilized a small sample size, which might impair the generalizability of the findings in other settings. The measurement of some variables is prone to recall bias. Though it is challenging to entirely avoid recall bias of such historical events using a cross-sectional design, we attempted to mitigate this bias by providing training to the data collectors to phrase questions correctly, using prompts related to historical events, and allowing sufficient time for participants to think and respond. Finally, the limited funding did not allowed to reach all paediatric service wings like Intensive Care unit (ICU).

## Conclusion and recommendation

This study revealed that 1 in 5 children and adolescents have an elimination disorder. Child age, large family size four and above, family history of elimination disorder, child and adolescents emotional and hyperactive problems, child and adolescents had bad parenting practices and low toilet training skill were identified as significant factors associated with elimination disorder. Therefore, as a paediatric public health issue, elimination disorder calls for intervention at all levels, including preventative, etiological, therapeutic, and curative. Furthermore, early toilet training, supportive parenting practices, screening for children's and adolescents’ behavioral problems, and elimination disorders need attention to reduce the effect of the problem.

## Data Availability

The datasets used and/or analyzed during the current study are available from the corresponding authors on reasonable request.

## Abbreviations

ABH: Abnormal Bowel Habit
APQ: Alabama Parenting Questionnaire
CI: Confidence Interval
CSTQ: Child Trauma Screening Questionnaire
DSM-5: Diagnostic and Statistical Manual of Mental Disorders Fifth Edition
DSSDES: Development of Symptom Score for Dysfunctional Elimination syndrome
DVISS: Dysfunctional Voiding and Incontinence Symptoms Score
ED: Elimination Disorder
IRB: Institutional Review Board
OR: Odds Ratio
PQ_EnU: Parental Questionnaire Enuresis/Urinary Incontinence
SDQ-PR: Strength and Difficulty Questionnaire Parent Report
WSUCSH: Wolaita Sodo University Comprehensive Specialized Hospital
